# Early Incarcerated Hernia at an 8-mm Robotic Port Site After Robot-Assisted Ileocecal Resection: A Case Report

**DOI:** 10.7759/cureus.109527

**Published:** 2026-05-23

**Authors:** Tenshi Makiyama, Atsushi Hirata, Tadashi Shiraishi, Koichiro Okada, Tetsuro Maruyama, Toru Tochigi, Gaku Ohira, Michihiro Maruyama

**Affiliations:** 1 Department of Frontier Surgery, Graduate School of Medicine, Chiba University, Chiba, JPN

**Keywords:** 8-mm port-site hernia, cancer colon, ileocecal resection, port-site hernia, robotic colorectal surgery, trocar-site hernia

## Abstract

Port-site hernia (PSH) after minimally invasive surgery is uncommon but can lead to bowel incarceration. Although fascial closure is generally recommended for port sites measuring 10 mm or larger, closure of 8-mm robotic port sites remains controversial. A 73-year-old Japanese woman with ascending colon cancer (cT2N0M0) underwent robot-assisted ileocecal resection with D3 lymph node dissection. She had a thin body habitus (height, 146 cm; weight, 33 kg; body mass index, 15.5 kg/m^2^). One 12-mm umbilical port, four 8-mm robotic ports, and one 5-mm assistant port were used. The umbilical mini-laparotomy wound was closed with peritoneal and fascial sutures, whereas the 5-mm and 8-mm port sites were closed with dermal sutures alone. On postoperative day 5, she developed vomiting and abdominal distension. Ultrasonography showed a small-bowel protrusion into the subcutaneous space at a left lower abdominal 8-mm robotic port site. Manual reduction was initially successful, but contrast-enhanced computed tomography on postoperative day 8 showed recurrent incarceration, and emergency surgery was performed. Richter-type small-bowel incarceration was identified at the 8-mm fascial defect without bowel necrosis. The bowel was reduced, and the peritoneum and fascia were closed. She recovered uneventfully and had no recurrent PSH at 30 months. Although available data do not support routine closure of all 8-mm robotic ports, selective fascial and peritoneal closure may be considered in selected high-risk patients, such as older or thin patients with potentially fragile abdominal walls.

## Introduction

Port-site hernia (PSH) is an uncommon but clinically important complication after minimally invasive surgery, as even a small fascial defect may lead to bowel incarceration and strangulation. PSH may occur when the fascial or peritoneal defect created by trocar insertion remains open or is enlarged by factors such as trocar manipulation, increased intra-abdominal pressure, or inadequate closure of the deep layers. Therefore, PSH should be regarded primarily as a trocar- or access-related complication, rather than a complication specific to a particular organ or resection procedure. In conventional laparoscopic surgery, PSH is mainly associated with port sites measuring 10 mm or larger, for which fascial closure is generally recommended [1,2]. However, with the increased use of robotic surgery, 8-mm ports have become standard in many procedures. Although large observational and prospective studies indicate a very low overall incidence of PSH at 8-mm port sites [3,4], symptomatic 8-mm PSH has been reported after robotic sigmoid or colon resection, including cases requiring bowel resection [5-7]. Similar cases have also been reported after robotic procedures in other surgical fields, as summarized in the Discussion. Therefore, the optimal management, including routine closure, of 8-mm robotic port sites, remains controversial.

Here, we report a case of early Richter-type incarcerated hernia at an 8-mm robotic port site after robot-assisted ileocecal resection, with recurrent incarceration after initial manual reduction. We also review previously reported cases of 8-mm robotic PSH to clarify the clinical features and potential preventive considerations of this rare complication.

## Case presentation

A 73-year-old Japanese woman (height, 146 cm; weight, 33 kg; body mass index, 15.5 kg/m^2^) was referred to our department after further evaluation of an abnormal chest shadow incidentally revealed abnormal uptake in the ascending colon on positron emission tomography-computed tomography. Further investigation led to a diagnosis of ascending colon cancer (cT2N0M0), clinical Stage I, according to the Japanese Classification of Colorectal, Appendiceal, and Anal Carcinoma [8]. She had no significant comorbidities, except for a history of surgery for uterine fibroids. Although her body mass index was low, oral intake was maintained preoperatively, and no specific nutritional intervention beyond routine perioperative management was performed. Robot-assisted ileocecal resection with D3 lymph node dissection was planned.

Surgery was performed as planned using the da Vinci Xi Surgical System (Intuitive Surgical, Sunnyvale, CA, USA), and robot-assisted ileocecal resection with D3 lymph node dissection was carried out. Six ports were placed as shown in Figure 1, including one 12-mm umbilical port, four 8-mm robotic ports, and one 5-mm assistant port. The specimen was extracted through a small extension of the umbilical 12-mm port site. The umbilical mini-laparotomy wound was closed with peritoneal and fascial sutures followed by subcuticular dermal closure, whereas the 5-mm and 8-mm port sites were closed with dermal sutures alone. During adhesiolysis of small-bowel adhesions in the pelvis, arterial bleeding from the mesentery occurred and was controlled with clips. There was no trocar slippage, dislodgement, or reinsertion during the procedure. However, the pelvic adhesiolysis and hemostatic maneuvers may have involved angled or repeated instrument manipulation. No intra-abdominal drain was placed. The total operative time was 2 h 52 min, console time was 65 min, and blood loss was 500 mL. Pathological examination revealed an ascending colon adenocarcinoma measuring 36 x 26 mm, with a final diagnosis of pT2N0M0, pathological Stage I [8].

**Figure 1:**
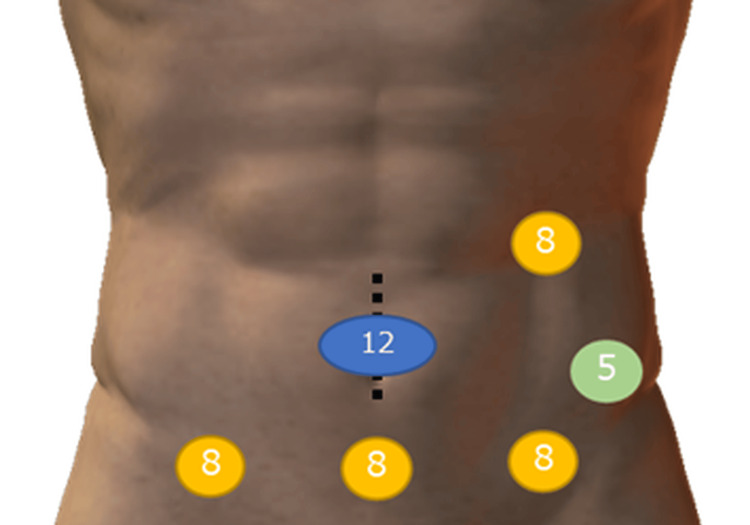
Port placement during robot-assisted ileocecal resection Six ports were placed, including one 12-mm umbilical port, four 8-mm robotic ports, and one 5-mm assistant port. The hernia developed at the left lower abdominal 8-mm robotic port site. This image was created by the authors using Microsoft PowerPoint for Microsoft 365 (Microsoft Corporation, Redmond, WA, USA).

The postoperative course was initially uneventful. Before the onset of vomiting and abdominal distension, she had no postoperative pneumonia, persistent cough, or other respiratory complications that could clearly have increased intra-abdominal pressure. However, on postoperative day 5, she began vomiting and developed abdominal distension. Abdominal ultrasonography demonstrated protrusion of the small bowel into the subcutaneous space at the left lower abdominal 8-mm robotic port site (Figure 2), and incarcerated PSH was diagnosed. Manual reduction was successfully performed. Because her symptoms temporarily improved and no signs of bowel ischemia were evident, elective repair was initially planned. However, contrast-enhanced computed tomography was performed on postoperative day 8 because recurrent incarceration was suspected after the initial manual reduction, rather than as part of routine postoperative surveillance. The CT scan showed recurrent incarceration at the same port site (Figure 3), without obvious radiological evidence of bowel ischemia. Therefore, emergency surgery was performed on the same day.

**Figure 2:**
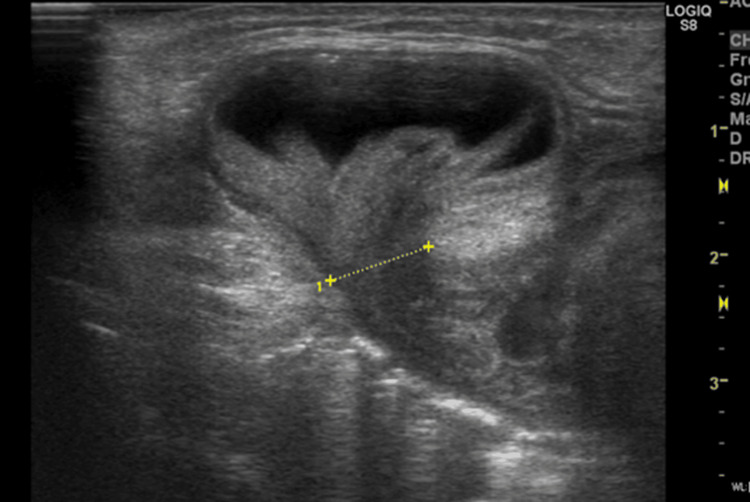
Abdominal ultrasonography findings Abdominal ultrasonography reveals protrusion of the small bowel through the left lower abdominal 8-mm robotic port-site defect into the subcutaneous space.

**Figure 3:**
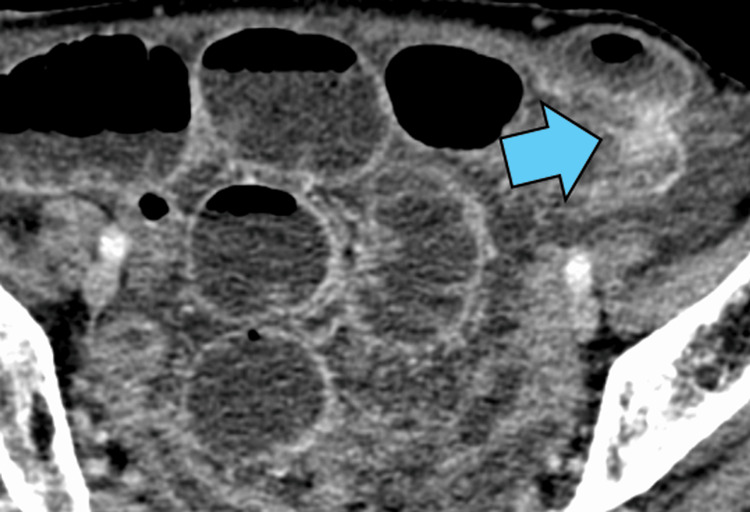
Contrast-enhanced computed tomography findings Contrast-enhanced computed tomography on postoperative day 8 reveals recurrent incarceration of the small bowel at the left lower abdominal 8-mm robotic port site after initial manual reduction.

During reoperation, the port-site wound was extended, and Richter-type incarceration of the small bowel through the 8-mm fascial defect was identified. The incarcerated bowel showed preserved color, and no irreversible ischemia or necrosis was observed. After incision of the fascia, the incarcerated bowel was reduced into the abdominal cavity, and the peritoneum and fascia were closed. The postoperative course after reoperation was uneventful, and she was discharged five days after reoperation. At the 30-day follow-up, laboratory tests showed no evidence of an inflammatory response, and imaging revealed no evidence of recurrent hernia. At 30 months after reoperation, she remained free from recurrent PSH.

## Discussion

In robotic surgery, 8-mm ports are routinely used, yet the need for fascial closure at these sites remains controversial. The present case indicates that 8-mm robotic PSH can cause early bowel incarceration requiring urgent reoperation. To place the present case in context, we conducted a focused, non-systematic review of English-language primary reports on PSH, with emphasis on 8-mm ports and abdominal or colorectal procedures. Relevant reports were identified by searching PubMed/PMC and major publisher websites for terms such as “robotic”, “8-mm port”, “PSH”, and “trocar-site hernia”; additional articles were identified by manual screening of reference lists. Representative reports most relevant to the present case are summarized in Table 1. These reports suggest that the overall incidence of PSH at 8-mm robotic port sites is very low. For instance, Damani et al. identified 15 cases of acute postoperative robotic PSH among 11,566 abdominal robotic procedures, 11 of which arose at 8-mm port sites [3]. Additionally, Cianfarani et al. found only one asymptomatic hernia among 513 8-mm trocars in a prospective series [4]. Milone et al. similarly reported only one case of PSH at an 8-mm trocar site among 320 robotic procedures in a retrospective cohort [9]. Among the reports in Table 1, symptomatic 8-mm robotic PSHs most frequently presented in the early postoperative period with small-bowel incarceration or obstruction. Further, several cases required urgent reoperation or bowel resection. Thus, although the overall incidence is low, the consequences can be substantial once herniation occurs.

**Table 1:** Representative English-language reports of 8-mm robotic port-site hernia PSH, port-site hernia; SBO, small-bowel obstruction; POD, postoperative day; RALS, robot-assisted laparoscopic surgery.

Study	Study design/setting	Onset/clinical presentation	Treatment/outcome	Key message
Damani 2021 [3]	Retrospective cohort; 11566 multispecialty abdominal robotic procedures	Acute postoperative PSH requiring reoperation within 30 days	15 acute PSHs; 3 bowel resections overall, including 2 among 8-mm PSHs	Large cohort showing extremely low incidence of acute 8-mm PSH; drain sites may require special consideration
Cianfarani 2024 [4]	Prospective cohort; 166 patients, 513 8-mm trocars in robotic abdominal wall surgery	One asymptomatic 1 × 1 cm supra-umbilical hernia detected at follow-up (median 14.5 months)	No repair	Prospective structured follow-up suggests an extremely low incidence without routine 8-mm fascial closure
Ahn 2024 [7]	Case report; robotic ileocecal resection	POD 11; 8-mm port-site incarceration	Manual reduction, then local closure; no bowel resection	Possible benefit of 8-mm port closure in selected cases
Liu 2018 [5]	Case report; robotic sigmoid resection	POD 7; closed-loop SBO from a right lateral 8-mm port	Laparoscopic reduction and fascial closure; no bowel resection	8-mm PSH may mimic prolonged ileus
Chang 2018 [6]	Case reports; 2 patients undergoing robotic colorectal surgery in obesity	POD 6–7; SBO due to incarcerated port-site hernia at an 8-mm robotic port	Laparoscopic reduction in both cases; 1 later required exploratory laparotomy and small-bowel resection	Possible contribution of obesity, long operative time, and port torque
Lim 2015 [10]	Case report; robotic low anterior resection	32 months; late hernia at a left mid-abdominal 8-mm drain site	Repair not detailed	Possible late presentation, particularly at drain sites
Tsu 2014 [13]	Case report/review; robotic prostatectomy	POD 4; SBO with bulge at a left lower 8-mm port (Spigelian-type)	Mini-laparotomy reduction and layered fascial repair; no bowel resection	Early SBO from a lateral 8-mm port; possible benefit of layered closure in patients with prior hernia, chronic cough, or constipation
Cho 2019 [17]	Case report; robotic cholecystectomy	POD 2; SBO from a left 8-mm port	Emergency surgery after failed conservative treatment; bowel resection and anastomosis	Very early severe ischemic presentation at a non-midline 8-mm working port
Micelli 2019 [16]	Case report; robotic colposacropexy	POD 3; SBO from a left lateral 8-mm port shortly after discharge	Laparotomy with 40-cm bowel resection	Potential for bowel loss and laparotomy
Uketa 2020 [18]	Case report; robotic cystectomy	POD 9; small-bowel incarceration at an 8-mm port	Emergency laparotomy with bowel resection and repair	Possible benefit of fascial closure in older patients with prior abdominal surgery
Ogasa 2020 [19]	Case report; robotic-assisted laparoscopic radical prostatectomy	POD 4; Richter-type incarceration at a right-hand 8-mm port	Emergency laparoscopic reduction and fascial/peritoneal closure; no bowel resection	Possible contribution of remote-center deviation and incomplete closure
Hong 2024 [21]	Two-case report/review; three-port robotic myomectomy	Early postoperative SBO from a left mid-axillary 8-mm drain site	Emergency exploration in both cases; bowel reduction and hernia repair without bowel resection	Drain-site vulnerability even in normal-BMI patients
Seike 2024 [22]	Two-case report; robotic nephrectomy and distal gastrectomy	POD 4–9; small-bowel incarceration at lateral 8-mm port	Laparoscopic or open release with fascial/peritoneal closure; no bowel resection	Thin patients with lateral ports; supports closure of all 8-mm ports after RALS

Within the colorectal literature, Ahn et al. reported an 8-mm PSH after robot-assisted ileocecal resection, similar to the present case; however, local closure after manual reduction was performed without bowel resection [7]. In contrast, the patient in the present case developed recurrent incarceration after initial manual reduction and required emergency reoperation, suggesting that temporary reduction does not necessarily represent definitive resolution. In addition, Liu et al. described a closed-loop small-bowel obstruction after robotic sigmoid resection [5], and Chang et al. reported two 8-mm robotic PSHs after colorectal surgery in patients with obesity, one of which required bowel resection [6]. Further, Lim et al. described a late incisional hernia at an 8-mm port site 32 months after robotic low anterior resection, indicating that colorectal 8-mm PSH may also present late [10].

The broader literature indicates that 8-mm robotic PSH is not limited to colorectal surgery. Representative reports from gynecologic, urologic, and general surgical fields have described 8-mm robotic PSH after procedures such as prostatectomy, hysterectomy, cholecystectomy, sacrocolpopexy, radical cystectomy, myomectomy, nephrectomy, and distal gastrectomy, often presenting early with evisceration, incarceration, or bowel obstruction [11-22]. Several patients required urgent surgery, and some underwent bowel resection because of ischemia [16-18,20]. A recent two-case report by Seike et al. highlighted incarcerated 8-mm PSH after robotic nephrectomy and distal gastrectomy and suggested direct-vision closure of 8-mm ports after robot-assisted laparoscopic surgery, particularly in thin patients with laterally placed ports [22]. Taken together, these reports indicate that 8-mm robotic PSH is rare but capable of causing substantial morbidity when diagnosis or repair is delayed.

Several factors may contribute to the development of PSH at 8-mm robotic port sites. Previously reported patient- and procedure-related factors include obesity, remote-center deviation, drain placement through the port, and inadequate closure of fascial or peritoneal defects [3,6,19-22]. Older age and thin body habitus, with potential abdominal wall vulnerability, as in the present case, may also be relevant [18,22]. In the present case, the patient’s very low body mass index suggested possible reduced muscle mass and abdominal wall vulnerability. However, formal sarcopenia assessment using skeletal muscle index, handgrip strength, or physical performance testing was not performed; therefore, sarcopenia could not be definitively diagnosed. Because low body mass index and possible frailty may increase vulnerability of the abdominal wall, preoperative nutritional and sarcopenia assessment may be useful in selected patients undergoing robotic surgery, although there is currently no direct evidence that short-term preoperative intervention prevents 8-mm PSH. Compared with conventional laparoscopy, robotic instruments may exert sustained torque on the abdominal wall, potentially enlarging a small fascial defect, even when the skin incision itself appears limited. In the present case, pelvic adhesiolysis and additional hemostatic maneuvers for mesenteric bleeding were required. Although no trocar slippage, dislodgement, or reinsertion occurred, repeated or angled manipulation of robotic instruments may have applied torque to the abdominal wall and enlarged the deep fascial or peritoneal defect without obvious enlargement of the skin incision. This mechanism remains speculative, and no causal relationship can be inferred from a single case.

At present, the available evidence does not support routine closure of all 8-mm robotic port sites. Prospective and retrospective studies indicate an extremely low incidence of trocar-site incisional hernia after 8-mm robotic access and suggest that routine fascial closure may not be necessary in all patients [3,4,9]. Nevertheless, the available case reports and cohort data, together with the present case, demonstrate that herniation at an 8-mm robotic port site can result in bowel incarceration, bowel resection, and urgent reoperation [3,5-7,10-23]. These findings support a selective rather than universal approach; fascial and peritoneal closure of 8-mm robotic port sites may be considered in selected high-risk situations, particularly in older or thin patients with potentially fragile abdominal walls, at drain sites, or when fascial defect enlargement is suspected. However, large studies establishing independent risk factors are needed.

## Conclusions

We report a case of early Richter-type incarcerated hernia at an 8-mm robotic port site after robot-assisted ileocecal resection. Although rare, PSH at an 8-mm robotic port site can cause bowel incarceration requiring urgent reoperation. The present case does not support routine closure of all 8-mm robotic ports; however, selective fascial and peritoneal closure may be considered when patient- or procedure-related risk factors are present, such as older age, very low body mass index, potentially fragile abdominal walls, drain placement, or suspected enlargement of the fascial defect.
